# Efficient 3-hydroxypropionic acid production by *Acetobacter* sp. CIP 58.66 through a feeding strategy based on pH control

**DOI:** 10.1186/s13568-021-01291-9

**Published:** 2021-09-20

**Authors:** Florence de Fouchécour, Anaïs Lemarchand, Henry-Éric Spinnler, Claire Saulou-Bérion

**Affiliations:** Université Paris-Saclay, AgroParisTech, INRAE, UMR SayFood, 78850 Thiverval-Grignon, France

**Keywords:** Organic acid, Biocatalysis, Fed-batch, Gompertz modelling, Synthon, Bioconversion

## Abstract

Acetic acid bacteria (AAB) can selectively oxidize diols into their corresponding hydroxyacids. Notably, they can convert 1,3-propanediol (1,3-PDO) into 3-hydroxypropionic acid (3-HP), which is a promising building-block. Until now, 3-HP production with AAB is carried out in batch and using resting cells at high cell densities (up to 10 g L^−1^ of cell dry weight). This approach is likely limited by detrimental accumulation of the intermediate 3-hydroxypropanal (3-HPA). Herein, we investigate an alternative implementation that allows highly efficient 3-HP production with lower cell densities of growing cells and that prevents 3-HPA accumulation. First, growth and 3-HP production of *Acetobacter* sp. CIP 58.66 were characterized with 1,3-PDO or glycerol as growth substrate. The strain was then implemented in a bioreactor, during a sequential process where it was first cultivated on glycerol, then the precursor 1,3-PDO was continuously supplied at a varying rate, easily controlled by the pH control. Different pH set points were tested (5.0, 4.5, and 4.0). This approach used the natural resistance of acetic acid bacteria to acidic conditions. Surprisingly, when pH was controlled at 5.0, the performances achieved in terms of titer (69.76 g_3-HP_ L^−1^), mean productivity (2.80 g_3-HP_ L^−1^ h^−1^), and molar yield (1.02 mol_3-HP_ mol^−1^_1,3-PDO_) were comparable to results obtained with genetically improved strains at neutral pH. The present results were obtained with comparatively lower cell densities (from 0.88 to 2.08 g L^−1^) than previously reported. This feeding strategy could be well-suited for future scale-up, since lower cell densities imply lower process costs and energy needs.

## Key points

Growing acetic acid bacteria can produce valuable 3-hydroxypropionic acid from 1,3-propanediol at pH = 5.0

A fed-batch process based on pH control was carried out to match the cell metabolic activity.

*Acetobacter* sp. CIP 58.66 is a relevant and efficient 3-HP producer.

## Introduction

Future petroleum shortage as well as environmental concerns have become major issues for the chemical industry to face in the upcoming decades. In order to ensure that these challenges are addressed and that companies remain competitive, public policies are pushing towards more bio-based processes. In 2015, bio-based chemicals represented only 6.8% of the overall manufacture of chemicals in the European Union (Piotrowski et al. 2018). Yet the European Union has set itself ambitious objectives through its Bio-based Industries Consortium (BIC). In particular, 30% of the production of chemicals in the European Union should become bio-based by 2030 (BIC 2012). In order to meet these objectives, new products and processes still need to be imagined or further optimized, so that they reach industrial scale and financial viability. Top value-added chemicals from biomass were listed by the US Department of Energy in order to focus research efforts on the most promising candidates (Bozell and Petersen 2010; Werpy and Petersen 2004). Among them is 3-hydroxypropionic acid (3-HP): its bifunctionality makes it a versatile platform molecule for further conversion into useful chemicals. Microbial production of 3-HP has been extensively studied and significant advances have been made (de Fouchécour et al. 2018; Kumar et al. 2013). However, some technical hurdles remain and bio-based 3-HP is not yet commercialized.

One of the dominant approaches so far has been 3-HP biosynthesis from glycerol, either through a coenzyme A-dependent or -independent metabolic route (de Fouchécour et al. 2018). In both cases, glycerol is first converted into 3-hydroxypropanal (3-HPA), which is subsequently oxidized into 3-HP or reduced into 1,3-propanediol (1,3-PDO). These oxidative and reducing pathways are in redox balance with one another: 1,3-PDO is an obligate co-product of the biosynthesis of 3-HP from glycerol. Consequently, it is of interest to implement a subsequent step for its conversion to 3-HP, so that the yield and the selectivity of the overall process are improved.

**Fig. 1:**
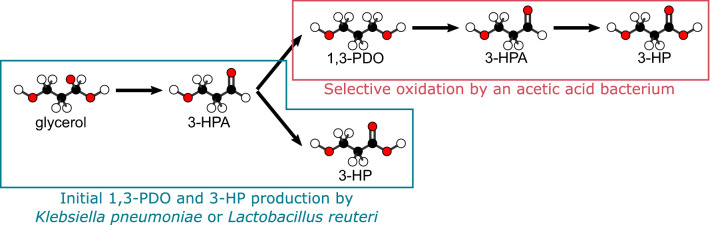
Schematic representation of the metabolic pathway for 3-HP production from glycerol

Acetic acid bacteria (AAB) have a double interest (i) they can selectively oxidise diols into hydroxycarboxylic acids (Füchtenbusch et al. 1998; Lynch et al. 2019); (ii) moreover, they have been selected, through evolution, to resist relatively high acid concentrations and low pH, because their activity acidifies their natural environments. In this light, 1,3-PDO conversion into 3-HP using AAB was recently investigated on a few occasions (Dishisha et al. 2015; Li et al. 2016; Zhao et al. 2015; Zhu et al. 2018). By analogy with ethanol oxidation into acetate, 3-HP synthesis from 1,3-PDO by these bacteria is thought to be a two-step pathway, set on the outer side of the membrane, with 3-HPA as an intermediate (Dishisha et al. 2015; Zhu et al. 2018). The AAB *Gluconobacter oxydans* was implemented, as part of multi-step processes where 1,3-PDO was first produced from glycerol by *Klebsiella pneumoniae* or *Lactobacillus reuteri* (Dishisha et al. 2015; Zhao et al. 2015). The pathway is presented on Fig. 1. The performances of immobilized *Acetobacter* sp. CGMCC 8142 for 3-HP production from 1,3-PDO were also evaluated (Li et al. 2016). These studies mainly focused on pH, temperature, and concentrations of substrate and biocatalyst as main parameters for process optimization. In all these studies, resting bacteria were operated in batch mode and showed promising results: 1,3-PDO was quantitatively converted to 3-HP, with little to no by-products, and high titres could be reached (up to 66.95 g L^−1^), thanks to the bacterial tolerance to acidic conditions. Acrylic acid was the only reported by-product of 1,3-PDO oxidation (Zhao et al. 2015). In their study, Dishisha et al. (2015) also observed transient 3-HPA accumulation, with a peak around 2 g L^−1^, which could be explained by an imbalance between the two successive enzymatic steps (Zhu et al. 2018). 3-HPA is a strong microbial inhibitor and could notably hinder performances of the process. Indeed, the accumulation observed by Dishisha et al. (2015) exceeded the minimum inhibitory concentrations (MIC) of 3-HPA that were estimated on *Escherichia coli* and ranged from 0.56 to 1.11 g L^−1^ (Cleusix et al. 2007).

Accumulation of 3-HPA will likely become a major limitation to the practical implementation of 3-HP production by AAB. In addition, previous studies used high AAB cell densities in their experiments: from 3.25 g of cell dry weight (DW) per litre (Dishisha et al. 2015), up to 10 g_DW_ L^−1^ (Zhao et al. 2015). Batch operation of such biocatalyst concentrations may contribute to fast 3-HPA accumulation, so future scale-up 3-HP production with AAB will necessitate alternative approaches. Genetic engineering work could help overcoming these challenges, for example by overexpressing the aldehyde dehydrogenase. Complementarily, a tailored bioprocess design can also contribute to increasing the production performance. This paper proposes a sequential process for efficient 3-HP production with the wild-type AAB *Acetobacter* sp. CIP 58.66 at low pH. First, the potential of the strain as 3-HP producer was verified. Preliminary tests were carried out in shake flasks, in order to identify the key limitations and the best suited operational conditions. When used as sole energy source, only low concentrations of 1,3-PDO could ensure sub-inhibitory 3-HPA levels and thus growth remained limited. A sequential process was then designed where the strain was first cultivated in batch mode on glycerol as energy source, then, at late exponential-phase, a fed-batch bioconversion of 1,3-PDO into 3-HP was started in order to keep a low 1,3-PDO concentration and to prevent high 3-HPA accumulation. Since the consumption of 1,3-PDO ultimately acidifies the medium due to 3-HP accumulation, a simple feeding strategy based on pH control was implemented for the fed-batch phase: 1,3-PDO was mixed with the base used for pH control in equimolar proportions. By doing so, it is expected that 1,3-PDO will be added continuously to the broth in the same amount than the amount of base required for 3-HP neutralization. A similar approach was already proven successful for lactic acid production by *Lactobacillus lactis* (Zhang et al. 2010). The present work investigates the performances and the advantages of such a strategy for 3-HP production by AAB at low pH which is a clear advantage for industrial production and is favourable to specific in situ product recovery (de Fouchécour et al. 2018). A kinetic analysis was carried out by fitting a modified Gompertz model to bacterial growth and 3-HP production kinetics. This allowed to estimate the specific production and growth rates and it contributed to a better description of the underlying physiological phenomena.

## Materials and methods

### Materials

The strain was purchased as *Acetobacter aceti* CIP 58.66 from the Biological Resource Center of Pasteur Institute (Paris, France). However, 16S rRNA gene analysis performed for this study revealed that the strain does not belong to the *aceti* species but to the *cerevisiae* species. The strain is thus referred to as *Acetobacter* sp. CIP 58.66 in this paper. Its 16S rRNA nucleotide sequence is available on the GenBank database with the accession number MZ242087.

Yeast extract and Bacto™ Peptone were obtained respectively from Organotechnie (La Courneurve, France) and BD-France (Le Pont de Claix, France). Monohydrate citric acid was purchased from Acros Organics (Geel, Belgium). Glycerol and trichloroacetic acid were acquired from VWR Chemicals (Leuven, Belgium). 1,3-PDO, K_2_HPO_4_, ammonium hydroxide and sulfuric acid were obtained from Sigma-Aldrich (Saint Louis, USA). 3-HP (30% w/v) was purchased from TCI Europe (Zwijndrecht, Belgium). Lastly, 3-HPA was chemically synthesized at URD Agro-Biotechnologies Industrielles (Pomacle, France) according to (Burgé et al. 2015).

### Preparation of the inocula

The strain was stored at – 80 °C in 20% (w/v) glycerol. For inocula preparation, 1 mL of stock culture was added to 25 mL of sterile, basal medium (5 g L^−1^ yeast extract, 8.71 g L^−1^ K_2_HPO_4_, 3 g L^−1^ peptone; pH was adjusted to 6.5 with a 5.5 mol L^−1^ H_2_SO_4_ solution) in 250 mL baffled shake flasks, and incubated for 63 h in an incubator shaker (Infors Multitron, Bottmingen, Switzerlan) at 30 °C and 200 rotations per minute (rpm). This inoculum was used directly for batch experiments in shake flasks. For bioreactor experiments, a second culture was carried out for volume expansion: 1 mL of the first culture was inoculated to 50 mL of sterile basal medium supplemented with glycerol (10 g L^−1^), in 500 mL baffled shake flasks. This culture was incubated 24 h at 30 °C and 200 rpm. Sterility was assured by autoclaving all medium-containing flasks at 120 °C for 20 min.

### Batch growth experiments in shake flasks

Batch cultures on 1,3-PDO were carried out in 500 mL baffled shake flasks containing 50 mL of sterile basal medium. Prior to inoculation, the medium was supplied with filter-sterilized 1,3-PDO, in order to reach concentrations of 5, 10 or 20 g L^−1^. Cultures were inoculated with an initial cell dry weight (DW) of 0.04 g_DW_ L^−1^. Cultures were then incubated at 30 °C and 200 rpm. Each condition was tested in duplicate. A control condition was also tested without any addition of 1,3-PDO to the basal medium.

Batch cultures on glycerol were performed similarly. Sterile basal medium was supplemented with 10 g L^−1^ of glycerol and a citrate–phosphate buffer was used (9 g L^−1^ K_2_HPO_4_ and 4.4 g L^−1^ citric acid). Initial pH was adjusted to 6.5, 5.0, 4.5 or 4.0, using H_2_SO_4_ (5.5 mol L^−1^). Each condition was tested in three replicates.

### Semi-continuous bioconversion experiments in a bioreactor

Semi-continuous bioconversions were carried out at 30 °C in a 3.6 L Labfors 4 bioreactor (Infors, Bottmingen, Switzerland), with an initial working volume of 1 L. The bioreactor was autoclaved with the medium at 120 °C during 20 min. Online measurements (temperature, pH, partial pressure of O_2_, stirring rate, air flow rate) were saved through Iris v.5 software (Infors). Partial pressure of O_2_ (pO_2_) and pH were monitored with 405-DPAS-SC and InPro 6800 probes respectively (Mettler Toledo). The pO_2_ probe was calibrated in the medium at 30 °C, just before inoculation. 100% and 0% values were calibrated by successively saturating the medium with dry air at 4 normal litres per minute (NL min^−1^) and with N_2_ (> 99%) at 1.5 bar, with a constant agitation speed of 400 rpm, during 20 min each. During bacterial cultures, agitation speed (100–800 rpm, Rushton turbine) and airflow rate (1–4 NL min^−1^) were automatically controlled in order to maintain pO_2_ above 40%.

**Fig. 2:**
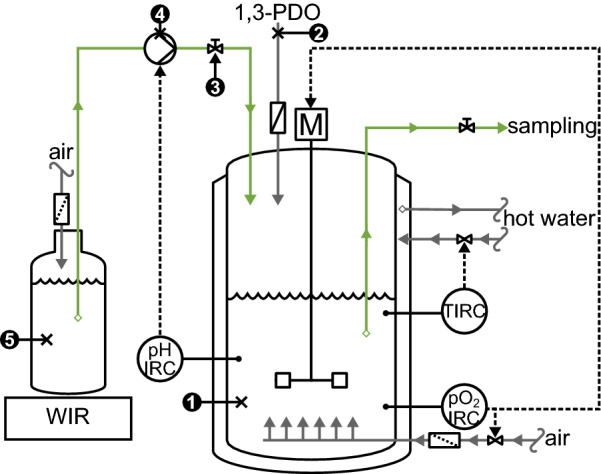
Schematic representation of the process. 1. Bioconversion medium. 2. Addition of filter-sterilized 1,3-PDO. 3. Manual valve opened when bioconversion starts. 4. Feed pump, activated depending on pH measurements. 5. Alkali and substrate mix. Solid arrows indicate fluid streams while dotted lines indicate electronic signals. M. Stirring motor. IRC. Indicator, recorder and controller of the concerned parameter [pH, pO_2_, temperature (T)]. WIR. Weight indicator and recorder

First, a growth phase on glycerol was carried out in batch mode. The initial medium (1 L) was composed of 10 g L^−1^ glycerol, 5 g L^−1^ yeast extract, 3 g L^−1^ peptone. pH was initially adjusted to 5.0 using H_2_SO_4_ (5.5 mol L^−1^) and then left uncontrolled during growth on glycerol. The bioreactor was inoculated so that the initial DW was around 0.02 g_DW_ L^−1^. Due to the inoculation, the initial pH rose to 5.2. Once the late exponential growth phase was reached, pH was adjusted to 4.0, 4.5 or 5.0 using H_2_SO_4_ (5.5 mol L^−1^). Filter-sterilized 1,3-PDO (5 mL) was then added to the medium in order to trigger bioconversion, and an equimolar solution of NH_4_OH and 1,3-PDO was plugged to the bioreactor, for both pH control and 1,3-PDO feeding purposes. Each condition was tested in duplicates. A schematic representation of the process is shown on Fig. 2.

### Analytical methods

#### Bacterial biomass measurements

Cell growth was monitored by off-line optical density (OD) measurements at 600 nm using an Evolution 201 spectrophotometer (ThermoScientific, Madison, USA). OD was correlated to cell dry weight (DW) using five dedicated shake flask cultures on glycerol. At 8, 24, 30 and 48 h of culture, 10 mL broth samples were taken and filtered on pre-dried PES filters (pore size: 0.2 µm). These filters were then dried in an oven at 90 °C for at least 24 h. Filters were weighted just before filtration and after drying, on a precision scale (Sartorius ED224S, Goettingen, Germany). DW was derived from the difference between the two weightings. Measurements were also performed with sterile, basal medium. The following relationship was established:

1 $${\mathrm{DW}}= 0.59 \cdot {\mathrm{ OD }}({\mathrm{R}}^2 = 0.96)$$

#### Chemical analysis

Glycerol, 1,3-PDO, 3-HP and 3-HPA were quantified by HPLC with a refractive index detector (Waters 2414, Guyancourt, France). 750 µL of each sample was mixed with 750 µL of a trichloroacetic acid solution (6% v/w) and put at 0 °C for at least 45 min, for protein precipitation. Samples were then centrifuged at 10,000*g* during 4 min at 4 °C. Supernatants were then filtered through nylon filters (pore size: 0.2 µm). Analysis was performed on an Aminex HPX-87H column (300 mm × 7.8 mm; Bio-Rad, Richmond, USA). 3-HPA was analyzed at a 0.6 mL min^−1^ flow rate at 35 °C, with 5 mmol L^−1^ H_2_SO_4_ as mobile phase. 1,3-PDO and 3-HP from samples not containing glycerol were analyzed in the same conditions, while samples containing glycerol were analyzed at a 0.4 mL min^−1^ flow rate at 65 °C, with H_2_SO_4_ at 0.5 mmol L^−1^ as mobile phase, allowing better resolution between 3-HP and glycerol peaks. Chromatograms were analyzed using Empower 3 software (Waters). Regarding 3-HP and 3-HPA, no high purity, analytical-grade product can be found commercially. Their quantifications are therefore likely to be subjected to higher measurements errors, which could explain why the overall carbon recovery of some experiments was found slightly above 100%.

#### Calculations

##### Working volume correction

In bioreactor experiments, despite the use of a condenser on the gas outlet pipe, a volume loss still occurred, due to dry air sparging and to long culture times. It was thus necessary to take this phenomenon into account in the analysis. For each experiment, the final working volume was measured and the overall volume loss was calculated proportionally to the cumulative air flow.

##### Kinetics analysis

Kinetics were analysed using the Gompertz model, as modified by Zwietering et al. (1990) (Eq. 2):

2 $$G\left(t\right)=a\cdot {\text{exp}}\left\{-{\text{exp}}\left[\frac{b\cdot e}{a} \left(c-t\right)+1\right]\right\},$$

where *e* represents exp(1), *t* is the time (h) and *a*, *b*, *c* are the parameters to be fitted. The model was first fitted to the natural logarithm of the relative population size ln(DW_t_/DW_0_). In this case, parameter *b* is the maximal specific growth rate µ_max_ (h^−1^). Results are given as parameter estimation ± standard error of estimation. Model fitting was validated by computing the Root Mean Square Error (RMSE). RMSE were normalised by the mean value of the experimental data, in order to compare them for different models.

The same model was also fitted to 3-HP concentrations: the time derivative of this is the volumetric productivity r_3-HP_ (g_3-HP_ L^−1^ h^−1^). Specific 3-HP productivities, q_3-HP_ (g_3-HP_ g_DW_^−1^ h^−1^), were estimated by first fitting dedicated Gompertz models to 3-HP and DW amounts in grams, in order to take into account the variation of volume. Then, using both fitted models, q_3-HP_ was computed using Eq. 3, with a 0.01 h time step:

3 $${q}_{\text{3-HP}}\left(t\right)= \frac{1}{DW(t)}\cdot \frac{d\text{3-HP}}{dt}.$$

Parameter optimisation was performed with the Levenberg–Marquardt least-squares method, using Python 3.6 and the “curve_fit” function from package scipy.optimize.

Because of a biphasic exponential growth on glycerol, specific growth rates were estimated for both phases separately by performing linear regression of ln(DW_t_/DW_0_) values against time. These regressions were done in Python 3.6, with function “OLS” from package statsmodel.api.

Moreover, number of generations N_g_ of a growth phase were computed as log_2_(DW_f_/DW_i_), where DW_i_ and DW_f_ are respectively the initial and the final DW values of the considered growth phase.

##### Statistical analysis

Results are all given as mean ± sample standard deviation, and the sample size is reminded as “n = ”. For n = 2, results are given as mean ± half the amplitude between the duplicates. Means were compared using the Fisher-Pitman permutation test, for which no distribution hypothesis is required. These tests were carried out with the “oneway_test” function (with “distribution” option set to “asymptomatic”) from “coin” package on R 3.3.1 software. Differences were considered significant when *p*-value was lower than 0.05.

## Results

### Effect of initial 1,3-PDO concentration in batch cultures

First, the ability of *Acetobacter* sp. CIP 58.66 to perform growth-coupled 1,3-PDO bioconversion was investigated. Growth was tested in shake flasks on a complex medium containing either 0, 5, 10 or 20 g L^−1^ of 1,3-PDO. To some extent, the basal medium without 1,3-PDO was sufficient to support the growth of the strain. Indeed, in absence of 1,3-PDO, growth was observed, with a final cell density of 0.18 g_DW_ L^−1^ (Table 1). When 1,3-PDO was added to the basal medium, changes in growth patterns were observed. Maximal growth rate µ_max_ was found significantly higher with 1,3-PDO than without (*p*-value = 0.01), but it was independent from the initial concentration. With 5 and 10 g L^−1^ initial 1,3-PDO, final cell densities were also higher compared to the control, whereas they were lower for 20 g L^−1^ initial 1,3-PDO (Table 1). These results show that *Acetobacter* sp. CIP 58.66 is able to use 1,3-PDO during its growth, either as carbon or energy source. Yet, the highest cell density was achieved with the lowest initial 1,3-PDO concentration, meaning that higher substrate levels cause inhibition either directly (substrate inhibition) or indirectly (product inhibition). Previous studies on 1,3-PDO bioconversion into 3-HP using AAB focused on resting cells only, and biomass evolution was not measured over time (Dishisha et al. 2015; Zhao et al. 2015). Therefore, this is the first report of 1,3-PDO affecting the growth of an AAB.

**Table 1:** Comparison of growth and bioconversion for different initial 1,3-PDO concentrations in shake flask cultures

	Initial 1,3-PDO concentration (g L^−1^)			
	0	5	10	20
Final DW (g L^−1^)	0.18 ± 0.01	0.33 ± 0.02	0.22 ± 0.03	0.12 ± 0.02
Number of generations (*N*_*g*_)	2.21 ± 0.06	3.17 ± 0.03	2.58 ± 0.09	1.72 ± 0.02
Final 1,3-PDO consumption (%)^a^	ND^b^	99.4 ± 0.7	53.7 ± 1.3	14.4 ± 1.4
Final 3-HP titre (g L^−1^)	ND^b^	6.02 ± 0.06	3.77 ± 0.61	2.14 ± 0.06
Final 3-HP yield (mol mol^−1^)	ND^b^	1.07 ± 0.03	0.59 ± 0.10	0.59 ± 0.01
Final 3-HPA titre (g L^−1^)	ND^b^	0.16 ± 0.00	1.06 ± 0.06	0.55 ± 0.03
Final 3-HPA yield (mol mol^−1^)	ND^b^	0.04 ± 0.00	0.21 ± 0.01	0.19 ± 0.01
Final pH	6.7 ± 0.08	4.29 ± 0.05	4.44 ± 0.13	5.11 ± 0.17
µ_max_ (h^−1^)	0.21 ± 0.01	0.28 ± 0.004	0.29 ± 0.01	0.27 ± 0.01
Normalised RMSE	2%	1%	2%	3%

Finals values are calculated at 30 h of culture

^a^Calculated as the percentage of 1,3-PDO initially supplied

^b^Was not calculated because no 1,3-PDO was supplied to the medium

The conditions with the best growth performances (5 g L^−1^ initial 1,3-PDO) was also the one displaying the best bioconversion performances: only in that condition was 1,3-PDO fully depleted. For 10 and 20 g L^−1^ initial concentrations, consumption dropped to 53.7% and 14.4%, respectively. This trend is consistent with the observations of Dishisha et al. (2015) on another AAB: in their study, resting cells of *Gluconobacter oxydans* consumed 95.3%, 86.1% and 31.3% of 5, 10 and 20 g L^−1^ of initial 1,3-PDO. Furthermore, full substrate depletion was also associated with the highest 3-HP yield and titre, as well as with the lowest level of 3-HPA production (Table 1). Contrarily, for 10 and 20 g L^−1^ initial 1,3-PDO, final 3-HP yields were lower: this was attributed to higher HPA accumulation.

These results show that AAB growth and 3-HP production is best achieved with a 5 g L^−1^ initial 1,3-PDO concentration. In this case, 1,3-PDO was quasi-quantitatively converted to 3-HP; substrate and 3-HPA inhibition were thus prevented. The almost quantitative production of 3-HP may suggest that 1,3-PDO is not used as carbon source for growth, but rather as energy source. In that case, the strain may have used another carbon source from the complex medium.

### Impact of initial pH on the growth on glycerol

Previous results showed that despite 1,3-PDO having a positive impact on the growth of *Acetobacter* sp. CIP 58.66, it could not ensure high biomass density. Growth was thus investigated on a separate substrate, namely glycerol. It was already used as growth substrate in other studies on 3-HP production with AAB (Dishisha et al. 2015; Li et al. 2016; Pyo et al. 2012; Zhao et al. 2015), but only few details were generally given on this step. In the present paper, four different initial pH were tested in shake flask, batch cultures of *Acetobacter* sp. CIP 58.66 on 10 g L^−1^ of glycerol (Table 2).

**Table 2:** Growth comparison of *Acetobacter* sp. CIP 58.66 with glycerol as substrate, at different initial pH

	Shake flask cultures^a^				Biocatalyst production^b^
Initial pH	4.0	4.5	5.0	6.5	5.0
DW (g_DW_ L^−1^)	0.14 ± 0.01	0.33 ± 0.04	1.14 ± 0.04	0.43 ± 0.02	0.88 ± 0.05
Glycerol consumption (%)^c^	1.0 ± 0.8	4.6 ± 1.9	16.9 ± 0.7	5.8 ± 0.6	18.6 ± 1.7
Biomass yield (${\text{g}}_{\text{DW}}\, {\text{g}}_{\text{glycerol}}^{-1}$)	ND^d^	0.83 ± 0.28	0.69 ± 0.05	0.76 ± 0.06	0.40 ± 0.05
Final pH	4.10 ± 0.02	4.58 ± 0.08	5.38 ± 0.03	6.55 ± 0.03	6.94 ± 0.13
µ_max,1_ (h^−1^)	0.10 ± 0.01	0.15 ± 0.004	0.18 ± 0.004	0.11 ± 0.004	0.24 ± 0.02
R^2^	0.94	0.98	0.99	0.98	0.88
µ_max,2_ (h^−1^)	0.03 ± 0.002	0.06 ± 0.002	0.09 ± 0.002	0.07 ± 0.001	0.18 ± 0.01
R^2^	0.92	0.97	0.99	0.99	0.96

^a^Comparison at 30.5 h of culture

^b^Values at 31 h of culture in bioreactor

^c^Calculated as the percentage of glycerol initially supplied

^d^Could not be calculated because no significant glycerol consumption occurred

When the initial pH was 4.0, glycerol remained unconsumed and biomass density remained low (0.14 g_DW_ L^−1^). This condition was thus considered too stressful and could not ensure high biocatalyst concentrations. In other conditions (i.e*.* initial pH of 4.5, 5.0 and 6.5), glycerol consumption occurred and higher biomass densities were reached (Table 2). The highest concentration (1.14 g_DW_ L^−1^) was obtained with an initial pH of 5.0. Even though glycerol consumption remained low at the time of comparison (30.5 h), full glycerol depletion was observed for all conditions after 115 h. In all instances, two distinct exponential growth phases were identified: a slope break appeared between 4 and 8 h of culture. The highest growth rates were observed with an initial pH of 5.0, which was also the only condition reaching late exponential phase by 30.5 h of culture. Since it led to the best growth performance (highest cell density, growth rates and biomass yield), pH 5.0 was the selected condition for the production of biocatalyst in a bioreactor, during the sequential process (*i.e.* biomass production directly followed by bioconversion).

### Biocatalyst production as part of a sequential strategy

First, we tested whether *Acetobacter* sp. CIP 58.66 could grow on 10 g L^−1^ glycerol in a bioreactor controlled at pH 5.0, while simultaneously converting 1,3-PDO into 3-HP. In this case, both growth on glycerol and 1,3-PDO conversion remained very limited (data not shown). Therefore, a sequential strategy was designed: biomass was first produced on glycerol in batch mode; once the late exponential phase was reached, 1,3-PDO was supplied in fed-batch mode. This section presents the first step (*i.e. *biocatalyst production) that was common to all sequential experiments.

**Fig. 3:**
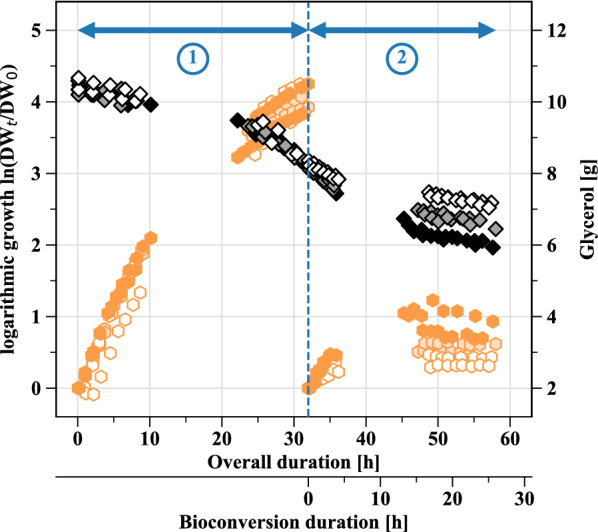
Growth of *Acetobacter* sp. CIP 58.66 and glycerol consumption during the sequential process. 1. Primary, batch growth on glycerol; this step is performed in the same conditions for all experiments. 2. Secondary growth during 1,3-PDO fed-batch bioconversion, at different pH (4.0, 4.5 or 5.0). For each growth phase, logarithmic growth is calculated on the basis of the initial DW of the concerned phase. For each pH condition, n = 2. The initial working volume was 1.02 L, and the final estimated volumes varied between 0.83 and 0.91 L, depending on replicates

Biomass was produced on the basal medium containing 10 g L^−1^ glycerol in a 3.6 L bioreactor. Initially, pH was set to 5.0 and it was then left uncontrolled. Results are shown in Table 2 and Fig. 3. Except for one of the six replicates, bacterial growth started without any latency. In all instances, late exponential phase was reached after 31 h of culture, with a 0.88 g L^−1^ DW concentration. Similarly to previous shake flask experiments, two distinct exponential growth phases could be detected. Furthermore, by 31 h of culture, glycerol consumption remained low (18.6% of initial glycerol). Since no buffer solution was added to the medium, pH rose up to 6.94. This rise in pH may be the cause of the slowing down of growth before full substrate depletion.

Growth on glycerol showed a satisfactory reproducibility: the coefficient of variation for final DW was 6%. It was thus considered that these six growth replicates constitute a consistent basis to compare the subsequent bioconversions at different pH.

### pH-based fed-batch bioconversion

**Fig. 4:**
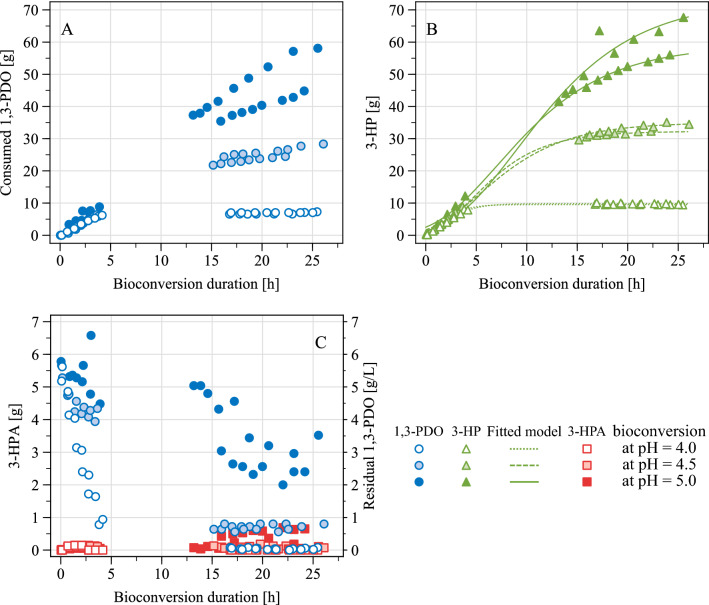
Bioconversion of 1,3-PDO into 3-HP by growing cells of *Acetobacter* sp. CIP 58.66 in a pH-based fed-batch, preceded by a first growth step on glycerol in batch mode. **A** Overall 1,3-PDO consumption. **B** Overall 3-HP production. **C** 3-HPA production and residual 1,3-PDO in the bioconversion broth. These bioconversions were launched after a 32 h-long growth on glycerol. Therefore *t* = 0 h on these graphs is equivalent to *t* = 32 h on Fig. 3. For each pH condition, n = 2. Depending on replicates, initial working volumes were estimated to be between 0.91 and 0.96 L, and final volumes between 0.83 and 0.91 L

Once produced as described in Sect. 3.2.2, biomass of *Acetobacter* sp. CIP 58.66 was used as biocatalyst for 1,3-PDO selective oxidation into 3-HP in presence of the residual glycerol. After growth on glycerol, pH was adjusted to the desired value with H_2_SO_4_ and the fed-batch bioconversion was triggered by adding 5 mL of 1,3-PDO as starter, and by plugging the feeding solution to the bioreactor. The latter solution was a 1:1 molar mix of ammonium hydroxide and 1,3-PDO in order to ensure both pH control and substrate feeding. In the first instance, bioconversion was tested at pH 5.0. A secondary exponential growth phase was observed, without any latency (Fig. 3). DW concentration further increased to 2.08 ± 0.04 g L^−1^ at a maximal growth rate of 0.16 ± 0.06 h^−1^. No significant difference in growth rates was evidenced when comparing with previous results for growth on glycerol alone or on 1,3-PDO alone. Glycerol was further consumed during 1,3-PDO oxidation (Fig. 3): its consumption increased from 18.6% at the beginning of bioconversion to 42.1% at 25 h of bioconversion. So it remains unclear whether this secondary growth can be attributed to the uptake of glycerol or 1,3-PDO, or both of them. Consumption of 1,3-PDO began as soon as it was added to the medium, without any latency (Fig. 4A). At 25 h of bioconversion at pH 5.0, a total of 51.45 ± 6.63 g of 1,3-PDO was consumed and converted quantitatively into 3-HP (Table 3; Fig. 4B). Within the first three hours, a transient accumulation of 3-HPA occurred, that peaked at 0.10 ± 0.01 g L^−1^. In one of the replicates, 3-HPA was later accumulated again, reaching a final concentration of 0.78 g L^−1^ (Fig. 4C).

**Table 3:** 1,3-PDO bioconversion characteristics of *Acetobacter* sp. CIP 58.66, after its growth on glycerol

	pH of bioconversion		
	5.0	4.5	4.0
Final DW (g L^−1^)	2.08 ± 0.04	1.58 ± 0.06	1.51 ± 0.04
Final *N*_*g*_^a^	1.21 ± 0.14	0.70 ± 0.03	0.54 ± 0.09
Final glycerol consumption (%)^b^	42.1 ± 0.6	36.0 ± 2.1	32.3 ± 1.8
Overall 1,3-PDO consumption (g)	51.45 ± 6.63	25.53 ± 1.07	7.15 ± 0.14
Final 3-HP titre (g L^−1^)	69.76 ± 6.00	36.48 ± 0.80	10.89 ± 0.65
Final 3-HP yield (mol mol^−1^)	1.02 ± 0.04	1.06 ± 0.05	1.11 ± 0.02
µ_max_ (h^−1^)	0.16 ± 0.06	0.13 ± 0.02	0.10 ± 0.03
Normalised RMSE for ln(DW_t_/DW_0_)	11%	8%	13%
r_3-HP,max_ (g_3-HP_ L^−1^ h^−1^)	3.9	2.7	2.4
q_3-HP,max_ (${\text{g}}_{\text{3-HP}}\,{\text{g}}_{\text{DW}}^{-1}\,{\text{ h}}^{-1}$)	2.0	2.0	2.3
Normalised RMSE for DW production	9%	6%	17%
Normalised RMSE for 3-HP production	5%	2%	3%

^a^Number of generations of the secondary growth phase

^b^Calculated as the percentage of glycerol initially supplied

The pH was successfully controlled at 5.0 during these bioconversions. However, 1,3-PDO was not kept at 5 g L^−1^ as intended: a slight decrease happened and the final concentration was 2.96 ± 0.79 g L^−1^ (Fig. 4C). In addition to 3-HPA accumulation in one replicate, two likely limiting factors for cells were identified: (i) high 3-HP concentrations, and (ii) oxygen limitation. Indeed, from 4.4 h of bioconversion, a drop in pO_2_ below the set point was observed, despite maximal stirring and air flow rates. In one instance, pO_2_ even dropped to 4% for 4 h before increasing again to the set point. Overall, the bioconversion process presented here achieved promising results, reaching the highest 3-HP titre with AAB. Also it is the first time growing AAB are used for 3-HP production instead of resting AAB. By doing so, the process could be performed with lower cell densities (from 0.88 to 2.08 g_DW_ L^−1^) as compared with the previous literature, where cell densities vary from 3.25 g_DW_ L^−1^ (Dishisha et al. 2015) to 10 g_DW_ L^−1^ (Zhao et al. 2015).

Given the promising results obtained at pH 5.0, the process was further tested for lower pH values during the bioconversion stage, in order to try to take advantage of the great resistance of AAB to acidic conditions. The selected pH values were 4.5 and 4.0: by doing so, the bioconversion performance can be compared for a pH above the pKa of 3-HP (pH 5.0), equal to it (pH 4.5), or lower to it (pH 4.0). Again, a secondary growth phase happened (Fig. 3). Yet, the lower the pH was, the lower were the growth rates, the final DW concentration, and the final glycerol consumption (Table 3). This is consistent with the results of shake flask cultures on glycerol, during which growth was more limited with initial pH values of 4.0 and 4.5 compared to 5.0. Concomitantly to this secondary growth phase, 1,3-PDO was consumed in all pH conditions and was quasi-quantitatively converted to 3-HP (Table 3). Similarly to bioconversions at pH 5.0, a slight 3-HPA accumulation occurred and peaked around 2 h of bioconversion, at sub-inhibitory levels, but no later 3-HPA accumulation was observed (Fig. 4). Overall, bioconversion performances were lower with lower pH: less 1,3-PDO was consumed and therefore less 3-HP was produced.

## Discussion

### Prevention of detrimental 3-HPA accumulation

Shake flask cultures on 1,3-PDO showed a drop in 3-HP yield for initial substrate concentrations higher than 10 g L^−1^ (Table 1), with 3-HPA accumulation being responsible for the lower 3-HP yield. Such a drop in 3-HP yield was already observed with immobilised cells of *Acetobacter* sp. CGMCC 8142, but only for substrate concentration above 50 g L^−1^, and the side-products were not mentioned (Li et al. 2016). A recent study on another AAB, *Gluconobacter oxydans* DSM 2003, suggested that the aldehyde dehydrogenase was the rate limiting enzyme of 1,3-PDO oxidation into 3-HP (Zhu et al. 2018). This could explain why 3-HPA accumulates for high initial substrate concentrations: in these cases substrate excess might have led to detrimental 3-HPA levels. Indeed, final 3-HPA concentrations were within the range of the MIC that was measured on the model organism *Escherichia coli*: from 0.56 to 1.11 g L^−1^ (Cleusix et al. 2007).

These observations highlight the importance of monitoring 3-HPA and preventing its accumulation, in order to ensure efficient 3-HP production with AAB. The study of Li et al. (2016) suggests that cell immobilisation on sodium alginate beads is a means of preventing 3-HPA accumulation, since 3-HP production yields remained between 90 and 100% with 1,3-PDO up to 50 g L^−1^. The present study offers an alternative for the prevention of 3-HPA accumulation: when 1,3-PDO levels were controlled under 5 g L^−1^ in the bioreactor, little to no 3-HPA accumulation occurred, and 3-HP was quantitatively produced up to 70 g L^−1^.

### Glycerol and 1,3-PDO metabolism of *Acetobacter* sp. CIP 58.66

To a large extent, the growth results obtained on glycerol in a bioreactor are comparable with those of Kylmä et al. (2004), who tested the growth of *Acetobacter aceti* on a mineral medium at pH 5.8, with glycerol as only substrate. In their experiment, at 32 h of culture, about 20% of glycerol was consumed and DW was around 0.5 g L^−1^. However, they observed lower specific growth rate (0.15 h^−1^) and biomass yield (0.16 g_DW_ g_glycerol_^−1^) than the ones reported in Table 2. These differences could be attributed to nutrient limitations because of the use of a minimal medium in the study of Kylmä et al.

Surprisingly, no product of glycerol catabolism could be detected in the present study. This is in contradiction with previous observations by Kylmä et al. (2004), who detected lactate and succinate (up to *ca.* 1.1 and 0.1 g L^−1^, respectively), when *A. aceti* was grown on 10 g L^−1^ glycerol. In these concentrations, lactate and succinate would have been detected in the conditions of analysis presented here. Besides, no medium acidification occurred in our experiments (Table 2). It was thus hypothesized that glycerol was fully oxidised into CO_2_, which was the main product from glycerol oxidation in the study of Kylmä et al. (2004). Moreover, maximal growth rates were lower than the ones determined in the presence of 1,3-PDO with the same initial pH (Table 1). This would suggest that glycerol and 1,3-PDO are metabolised in two distinct metabolic pathways by *Acetobacter* sp. CIP 58.66. This is consistent with studies of Kylmä et al. (2004) and Zhu et al. (2018): the former proposed two distinct pathways for glycerol and ethanol metabolism in *Acetobacter aceti*, while the latter showed that the AAB *Gluconobacter oxydans* oxidises 1,3-PDO through a pathway similar to the one of ethanol. Further investigations would be required in order to understand what triggered the secondary growth phase during bioconversion and what the contributions of these two metabolic pathways are. Two more factors are likely to have contributed to triggering the new growth phase: (i) pH adjustment prior to bioconversion; and (ii) ammonium hydroxide addition as pH-control agent, also potentially serving as nitrogen source.

### Balance in feeding solution for pH-based fed-batch

The pH-based fed-batch strategy was shown relevant for pH control and substrate feeding. However, for lower pH conditions, it appeared that 1,3-PDO levels decreased instead of keeping around 5 g L^−1^. 3-HP having a pKa of 4.51 (Lide 2005), the undissociated (or protonated) and dissociated (or anionic) forms are present in almost equal amounts for pH 4.5. For pH 5.0, the dissociated form is predominant over the other; while the undissociated form is predominant at pH 4.0. Therefore, even though 3-HP production was quite similar for all pH during the first 5 h of bioconversion (Fig. 4B), the lower the pH was, the more predominant was the protonated form. Because of its higher lipophilicity, the protonated form is usually considered as more toxic to cells than the anionic form (Trček et al. 2015). It enters the cell through passive diffusion in the membrane, thus leading to cytosolic acidification and to the disruption of the electro-chemical gradient. This could contribute to explain the shorter bioconversion duration at pH 4.0 compared to 5.0. Resistance mechanisms are then triggered in AAB, including efflux pumps (Nakano and Fukaya 2008). In particular, *A. aceti* IFO 3283 was shown to possess a proton motive force-dependent efflux system for acetic acid resistance (Matsushita et al. 2005). Despite a relative specificity for acetic acid, this system seemed to work with short-chain organic acids, and the presence of a respiratory substrate could enhance this activity. Since 1,3-PDO oxidation in AAB is associated to the electron transport chain (at least through the *adhB* subunit of the alcohol dehydrogenase) (Zhu et al. 2018), 1,3-PDO may be considered as a respiratory substrate. Existence of such an efflux system in *Acetobacter* sp. CIP 58.66 – with 1,3-PDO as respiratory substrate – could contribute to explaining the apparently paradoxical increase in specific productivities for the lowest pH (Table 3).

Furthermore, consumption of one equivalent of 1,3-PDO would lead to less important proton release for lower pH, due to the predominance of undissociated 3-HP. Consequently, smaller volumes of the feeding solution are needed for pH control. In our case, this means that there is also less 1,3-PDO supplied than consumed: the lower the pH, the greater the imbalance. This explains why 1,3-PDO could not be kept at 5 g L^−1^ as desired, and was even completely depleted after 10 h of bioconversion at pH 4.0. These results suggest that the ammonia to 1,3-PDO ratio in the feed should be determined specifically for each pH condition, in order to prevent the slowing down of the process. Adequate base-to-substrate ratio might help taking advantage of the strain’s great potentialities, even at low pH. Maintaining pH levels below the pKa of 3-HP would also be an advantage for its downstream recovery. Indeed, the most popular technique in the literature for 3-HP recovery is in situ product recovery by liquid–liquid reactive extraction during bioconversion (de Fouchécour et al. 2018). This technique requires that 3-HP is in its undissociated form to be extracted.

In conclusion, the designed process using wild-type *Acetobacter* sp. CIP 58.66 achieved high productivities and titres, with a quasi-quantitative molar yield, thus limiting accumulation of detrimental 3-HPA. These performances (titre and productivity) are the highest reported with AAB and they were comparable to those of the most efficient genetically engineered 3-HP producers. Moreover, it was possible at acidic pH (5.0) which limits the risk of contamination and is a significant advantage for industrial production. This point is also favourable to the in situ product recovery. These results also demonstrate that high bacterial densities are not required for 3-HP production by AAB, but that the substrate 1,3-PDO should be fed at a rate compatible with the biocatalytic activity. Moreover, this is the first report of 1,3-PDO affecting the growth of an AAB. Processes using obligate aerobes, such as *Acetobacter* sp. CIP 58.66, generally have high energy demands due to aeration (air compressors and stirring). Thus, achieving efficient 3-HP production with low cell densities may become an asset when considering scale-up. Key limitations were identified here, viz. dioxygen availability, low pH, and composition of the feeding solution. The proposed feeding strategy seems promising for its implementation in a complete integrated process for 3-HP production from glycerol.

## Data Availability

The datasets used in the current study are available from the corresponding author on reasonable request.

## Code availability

The code used for non-linear regression is available from the corresponding author on reasonable request.
